# Identification and Characterization of the First *Escherichia coli* Strain Carrying NDM-1 Gene in China

**DOI:** 10.1371/journal.pone.0066666

**Published:** 2013-06-10

**Authors:** Zhiyuan Liu, Wei Li, Jie Wang, Jian Pan, Shipeng Sun, Yanhua Yu, Bing Zhao, Yuzhi Ma, Tingju Zhang, Jie Qi, Guijian Liu, Fengmin Lu

**Affiliations:** 1 Guang'anmen Hospital, China Academy of Chinese Medical Sciences, Beijing, PR China; 2 Department of Microbiology, Center for Infectious Diseases, Peking University Health Science Center, Beijing, PR China; 3 Center for Infectious Diseases, Beijing You'an Hospital, Capital Medical University, Beijing, PR China; 4 Department of Clinical Laboratory, Beijing You'an Hospital, Capital Medical University, Beijing, PR China; Iowa State University, United States of America

## Abstract

New Delhi metallo-β-lactamase-1 (NDM-1), an acquired class B carbapenemase, is a significant clinical threat due to its extended hydrolysis of β-lactams including carbapenems. In this study, we identified the first confirmed clinical isolate of *Escherichia coli BJ01* harboring *bla*_NDM-1_ in China. The isolate is highly resistant to all tested antimicrobials except polymyxin. *bla*_NDM-1_, *bla*_CTX-M-57_, and *bla*_TEM-1_ were identified in the isolate. *bla*_NDM-1_ was transferable to *E. coli EC600* and *DH5α* in both plasmid conjugation experiments and plasmid transformation tests. *BJ01* was identified as a new sequence type, ST224, by multilocus sequence typing. Analysis of genetic environment shows complex transposon-like structures surrounding the *bla*_NDM-1_ gene. Genetic analysis revealed that the region flanking *bla*_NDM-1_ was very similar to previously identified NDM-positive *Acinetobacter* spp. isolated in China. The findings of this study raise attention to the emergence and spread of NDM-1-carrying *Enterobacteriaceae* in China.

## Introduction

Antimicrobial resistance is a growing global challenge to human health[1]. The emerging New Delhi metallo-β-lactamase (NDM-1), an acquired class B carbapenemase that was first clinically detected in a patient at a hospital in New Delhi, India, has brought up worldwide public attention again[2]–[4]. It confers resistance to a broad-spectrum of β-lactams, including carbapenems, which are the mainstream treatment for antibiotic-resistant bacterial infections[5]. Although current reports indicate that NDM-1 does not hydrolyze monobactams, most of NDM-1-carrying strains also express enzymes that could hydrolyze monobactams, making NDM-1-producers very difficult to control[6]. The rapid dissemination of NDM-1-producing gram-negative species also contributes to this major concern of public health. It has been reported in >50 countries across five continents, in the last 2 years[2], [4].

NDM-1 is detected mainly in Escherichia coli and Klebsiella pneumonia but occasionally in Klebsiella oxytoca, Citrobacter freundii, Morganella morganii, Providencia spp., Enterobacter cloacae, Proteus spp., Acinetobacter baumannii, Stenotrophomonas maltophilia and Pseudomonas aeruginosa [3], [7], [8]. To our knowledge, NDM-1-producing strains have been seen only in Acinetobacter spp. [9]–[12] and Enterococcus faecium[13] in China. There is no evidence of the emergence of NDM-1-producing Enterobacteriaceae in China at this point, although it was found in the stool samples of a 1-year-old infant and his mother in Hong Kong who once traveled to and were hospitalized in Hunan Province, China[14]. In the present study, we report the first confirmed case of NDM-1-producing E. coli infection in Beijing, China.

## Materials and Methods

### Patient Information

A 75-year-old Chinese patient with diabetes, who came from the Anhui province in southern China, was admitted for diabetes-related foot complications comprising a swollen peak of the left foot and ulceration of the fourth and fifth toes on September, 2011. The NDM-1-producing *E.coli*, *BJ01*, strain was isolated from the ulcer secretion. Since the strain was found to be resistant to almost all tested antibiotics, a combination of intravenous levofloxacin and etimicin was given empirically. Amputation of the fifth metatarsal bones and an incisional drainage operation were performed. The wound started healing nicely. The patient was discharged from the hospital one month after admission. Neither the patient nor the patient's family members had a history of traveling to the Indian subcontinent or other countries. The patient in this manuscript has given written informed consent, as outlined in the PLOS consent form to publication of their case details.

### Bacterial Isolate and Phenotypic Screening for MBL

The clinical isolate *BJ01* of the metallo-β-lactamase(MBL)-producing *E. coli* was derived from the ulcer secretion of the patient collected on the admission day. The strain with same phenotype was still positive on day 5 after the operation. The identification and drug susceptibility of the bacteria were tested by using Vitek 2 Compact (bioMerieux, Marcy I''Etoile, France). Minimal inhibitory concentrations (MICs) of 18 antibiotics and combinations of antimicrobials were determined by broth microdilution method in accordance with the guidelines of the Clinical and Laboratory Standards Institute (CLSI)[15]. Most of antimicrobial agents were obtained from the National Institute for the Control of Pharmaceutical and Biological Products (NICPBP), except for imipenem (Merck Sharp & Dohme, Whitehouse Station, USA), meropenem (Dainippon Sumitomo Pharma, Chuo-ku, Japan) and polymyxin (Sigma, St. Louis, USA). *E.coli* strain ATCC25922 was used as a quality control for MIC determination. MBL production was tested by comparing diameters of the zones of inhibition for meropenem (10 µg) and imipenem (10 µg) on Mueller-Hinton agar (MHA) versus MHA impregnated with EDTA, an MBL inhibitor. EDTA-impregnated MHA was prepared by spreading 2 mL of 5 mM EDTA on MHA [16].

### Polymerase Chain Reaction (PCR) Amplification for the Detection of MBL and other β-lactamase genes

The primers used (NDM-F1, 5′-GGCGGAATGGCTCATCACGA-3′; NDM-R1, 5′-CGCAACACAGGCCTGACTTTC-3′) were those recommended by the Chinese Center for Disease Control and Prevention. For the specific detection of *bla*_NDM-1_, primers NDM-F2 (5′-ATGGAATTGCCCAATATTATGC-3′) and NDM-R2 (5′-TCAGCGCAGCTTGTCGGCCAT-3′) were used to amplify the entire bla_NDM-1_ gene. At that time, we performed molecular testing for other MBL, including *bla*_VIM_, *bla*_IMP_, *bla*_GIM_, *bla*_SPM_, and *bla*_SIM_ [17], [18]. PCR was also undertaken for other β-lactamase genes, including *bla*_TEM_, *bla*_SHV_, *bla*_CTX-M_, and *bla*_OXA-1_ [19]. *bla*_CTX-M_ was further identified using the specific primers for the CTX-M group 1 (*CTX-M-1grpF*, 5′-CCAGAATAAGGAATCCCATG-3′; *CTX-M-1grpR*, 5′-GCCGTCTAAGGCGATAAAC-3′), CTX-M group 2 (*CTX-M-2grpF*, 5′-ATGATGACTCAGAGCATTCG-3′; *CTX-M-2grpR*, 5′-TGGGTTACGATTTTCGCC-3′), and the CTX-M group 9 (*CTX-M-9grpF*, 5′- ATGGTGACAAAGAGAGTGCA-3′; *CTX-M-9grpR*, 5′-CCCTTCGGCGATGATTCTC-3′). All positive PCR products were sequenced and the sequencing results were compared with previously reported sequences available in GenBank.

### Southern Blot

Southern blot was performed by using DIG High Prime DNA Labeling and Detection Starter Kit II (F. Hoffmann-La Roche Ltd., Basel, Swiss) following manufacturer's protocol. Briefly, genomic DNA was digested with restriction enzyme *HindIII* and *BamHI*. The digested DNA fragments were transferred to nylon membrane (Pharmacia & Biotech, Piscataway, USA), and hybridized with digoxigenin-labeled *bla*_NDM-1_-specific probes and anti-DIG-AP antibody. The membrane was visualized by applying chemiluminescent substrate CSPD and X-ray exposure.

### Conjugation and Transformation

The transfer of carbapenem resistance was tested using a conjugation test (broth mating method). *E. coli EC600* (LacZ^−^ Nal^r^ Rif^r^) was used as the recipient strain. Overnight cultures of the donor strain (300 µL) and recipient strain (100 µL) were mixed with 600 µL of fresh Mueller-Hinton broth and were incubated overnight at 35°C. The mixture was then inoculated on MHA plates containing rifampin (Sigma, St. Louis, USA, 100 µg/mL) plus ceftriaxone (F. Hoffmann-La Roche Ltd., Basel, Swiss, 4 µg/mL) for 24 h at 35°C. The bacterial colonies were then transferred to a plate containing rifampin (Sigma, St. Louis, USA, 100 µg/mL) plus imipenem (Merck Sharp & Dohme, Whitehouse Station, USA, 4 µg/mL) for 24 h at 35°C by replica plating, which maintains the original colony pattern.

Plasmid DNA was extracted using a Qiagen Mini Kit (Qiagen, Hilden, Germany) and was transformed into *E. coli DH5α*-competent cells (TAKARA, Shanghai, China). Transformants were selected on MHA plates containing IPM (4 µg/mL).

The identification and susceptibility of the transconjugant and transformant were confirmed via using the VITEK 2 system. PCR analysis was used to determine the presence of carbapenemase genes.

### Multilocus Sequence Typing (MLST) and Sequencing of Genetic Environment

MLST with seven housekeeping genes (*adk, fumC, gyrB, icd, mdh, purA*, and *recA*) was performed according to the protocol described on the *E. coli* MLST website (http://mlst.ucc.ie/mlst/dbs/Ecoli). The regions flanking the *bla*_NDM-1_ gene were sequenced by primer walking strategy, starting from each end of the *bla*_NDM-1_ gene in the *DH5α_NDM-1_* transformant. The plasmid containing *bla*_NDM-1_ gene was used as a template for primer walking. The first set of primers was designed targeting each end of the *bla*_NDM-1_ sequence (5′-GGTCGCCAGTTTCCATTTGC-3′ and 5′-TGCCGACACTGAGCACTAC-3′). The amplification products were sequenced. Then new primers complementary to the known sequence were designed and synthesized for sequencing the unknown DNA sequence next to it. Totally, 13 pairs of primers were synthesized. Sequence files were assembled and aligned by using ContigExpress software.

## Results

### Antimicrobial Susceptibility of *E. coli BJ01* Strain

**Figure 1:**
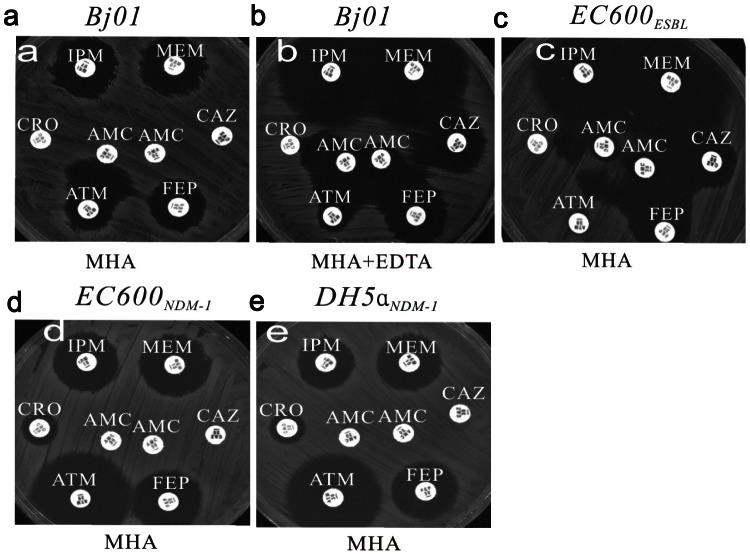
Phenotypic detection of carbapenemases and extended-spectrum β-lactamase (ESBL) in *E. coli BJ01*. **a.** Antibacterial susceptibility testing for *BJ01* on the Mueller-Hinton agar (MHA). **b.** Antimicrobial susceptibility testing for *BJ01* on MHA impregnated with 2 mL of ethylenediaminetetraacetic acid (EDTA) 5 mM. **c.** Antimicrobial susceptibility testing for the *EC600* transconjugant (*EC600_ESBLs_*). **d.** Antimicrobial susceptibility testing for the *EC600* transconjugant (*EC600_NDM-1_*). *EC600_NDM-1_* was resistant to imipenem, meropenem, and cephalosporins except for ATM and showed no synergy between AMC and cephalosporins. **e.** Antimicrobial susceptibility testing for the *DH5α* transformant (*DH5α_NDM-1_*). The phenotype was the same as that of *EC600_NDM-1_*. IMP, imipenem; MEM, meropenem; CRO, ceftriaxone; AMC,amoxicillin-clavulanic acid; CAZ, ceftazidime; ATM, aztreonam; FEP, cefepime.

*E. coli BJ01* isolated from the patient displayed high resistance to all tested antibiotics, including ampicillin, ampicillin-sulbactam, cefazolin, aztreonam, ceftriaxone, cefepime, imipenem, meropenem, ceftazidime, piperacillin-tazobactam, cefotetan, tobramycin, gentamicin, ciprofloxacin, levofloxacin, amikacin and trimethoprim-sulfamethoxazole, except polymyxin, which showed bacteriostatic activity to *BJ01* (MIC 1 µg/ml) (**Table 1**, **Fig. 1**). On MHA, amoxicillin-clavulanic acid showed no synergy with cephalosporins except for aztreonam since metalloenzymes do not hydrolyze aztreonam (**Fig. 1a**). On MHA containing EDTA, metalloenzyme activity was inhibited, the diameters of the inhibition zones for MEM (meropenem) and IPM (imipenem) were both 18 mm greater than on MHA without EDTA, and synergistic function was observed between amoxicillin-clavulanic acid and ceftazidime, ceftriaxone, aztreonam and cefepime (**Fig. 1b**). Above findings are suggestive of ESBL (extended-spectrum β-lactamase) production in *E. coli BJ01* [20].

**Table 1:** Antibiotic susceptibility profiles of different strains (MIC (µg/mL)).

	MIC(µg/mL)					
	*BJ01*	*EC600* recipient	*EC600_ESBLs_* conjugant	*EC600_ NDM-1_* conjugant	*DH5α* recipient	*DH5α_NDM-1_* transformant
Ampicilin	>256	2	>256	>256	2	>256
Ampicillin-sulbactam	>256	2	>256	>256	2	>256
Cefazolin	>256	2	>256	>256	1	>256
Aztreonam	256	0.25	256	0.50	0.50	0.50
Ceftriaxone	>256	0.25	256	256	0.25	256
Cefepime	256	≤0.125	128	128	≤0.125	128
Imipenem	32	≤0.125	≤0.125	32	≤0.125	32
Meropenem	32	≤0.125	≤0.125	16	≤0.125	32
Ceftazidime	>256	0.25	16	>256	0.25	>256
Piperacillin-tazobactam	256	1	1	128	1	128
Cefotetan	256	0.5	0.5	128	1	128
Tobramycin	256	0.25	128	0.5	0.25	0.5
Gentamicin	256	0.25	128	0.25	0.25	0.25
Ciprofloxacin	32	0.25	1	0.25	0.25	0.25
Levofloxacin	16	1	1	0.5	0.25	0.25
Amikacin	>256	0.25	256	0.50	0.50	0.50
Trimethoprim-sulfamethoxazole	>32/608	0.5/9.5	0.5/9.5	0.5/9.5	0.5/9.5	0.5/9.5
Polymyxin	1	0.5	0.5	0.5	0.5	0.5

MIC, minimum inhibitory concentration; ESBL, extended-spectrum β-lactamase; NDM, New Delhi metallo-β-lactamase-1.

### Transfer of Antibiotic Resistance

In the conjugation experiments, two resistant phenotypes of *E. coli EC600* transconjugants (*EC600_ESBL_ and EC600_NDM-1_*) were found by using replica plating methods, presenting with different drug resistant patterns (**Table 1**). The IMP (imipenem) and MEM (meropenem) sensitivity of *EC600_ESBL_* and the synergy between AMC (amoxicillin-clavulanic acid) and FEP (cefepime), CAZ (ceftazidime), CRO (ceftriaxone), ATM (aztreonam) on the strain suggested ESBL production (**Fig. 1c**). *EC600_NDM-1_* grew on both MHA containing rifampin plus imipenem and MHA containing rifampin plus ceftriaxone. While, *EC600_ESBL_* grew only on MHA containing rifampin plus ceftriaxone. This indicates that the carbapenemase gene and ESBL gene may be on different plasmids and therefore transferred independently. *EC600_ESBL_* had the same resistance phenotype as *BJ01* on MHA containing EDTA because the carbapenemase in *BJ01* was inhibited on MHA impregnated with EDTA. (**Fig. 1c**). The *EC600_NDM-1_* transconjugant was resistant to carbapenems and cephalosporins but sensitive to aminoglycosides, fluoroquinolones, and aztreonam, which indicated that plasmid harboring *bla*_NDM-1_ did not carry other ESBL, aminoglycoside-resistance, or quinolone-resistance genes (**Fig. 1d**). The prepared plasmid was transformed into *E. coli DH5α*. Antimicrobial susceptibility profiles of the *DH5α_NDM-1_* transformant exhibited antibiotic sensitivities similar to the *EC600_NDM-1_* isolate (**Fig. 1e** **;** **Table 1**).

### Detection of Drug Resistance Genes

**Figure 2:**
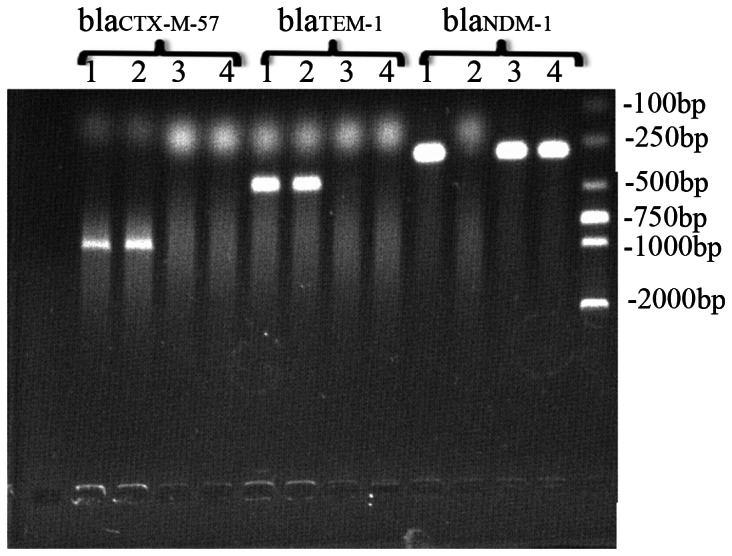
Polymerase chain reaction-amplified CTX-M-57, TEM-1, and NDM-1 genes in BJ01 (1), EC600_ESBL_ (2), EC600_NDM-1_ (3), and DH5α_NDM-1_ (4). CTX-M-57 and TEM-1 were detected in BJ01 and EC600_ESBL_; bla_NDM-1_ was detected in BJ01, E600_NDM-1_, and DH5α_NDM-1_.

**Figure 3:**
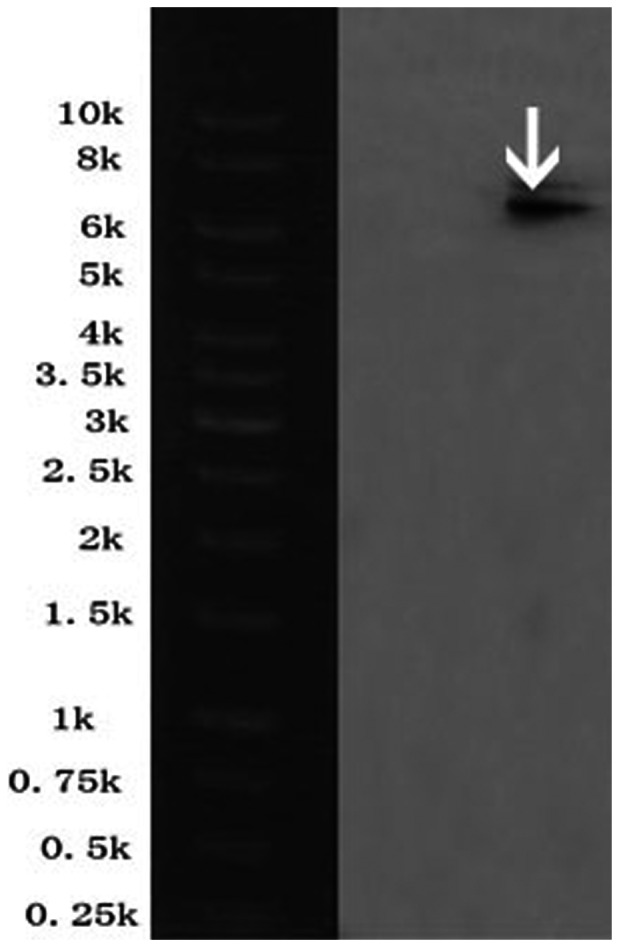
Southern blot hybridization on *bla*_NDM-1_ gene of *BJ01*. The band marked with the white arrow indicated positive signals by Southern blot hybridization with the specific NDM-1 probe.

By PCR and sequencing, we detected the presence of MBL genes in *BJ01* strain. The *bla*_NDM-1_, *bla*_TEM-1_ and *bla*_CTX-M-57_ genes detected (**Fig. 2**), but not any other MBL genes (*bla*_VIM_, *bla*_IMP_, *bla*_GIM_, *bla*_SPM_, and *bla*_SIM_) or β-lactamase genes (*bla*_SHV_ and *bla*_OXA-1_). The presence of *bla*_NDM-1_ gene was further confirmed by Southern blot (**Fig. 3**). CTX-M-β-lactamases can be divided into five groups based on their amino acid sequence identities. Group I includes CTX-M-1, -3, -10 to -12, -15, -22, -23, -28, -29, etc. CTX-M-57 is a group I CTX-M and shared 99% amino acid identity with CTX-M-15[21], [22], which is one of the most common types of ESBL found in bacterial isolates[23]. The sequences of the *bla*_NDM-1_, *bla*_TEM_, and *bla*_CTX-M-57_ genes were analyzed and deposited in GenBank under accession numbers HQ603057, JX036279, and JX036278. Only *bla*_TEM-1_ and *bla*_CTX-M-57_ were detected in the *EC600_ESBL_* transconjugant and only *bla*_NDM-1_ was detected in the *EC600_NDM-1_* transconjugant and *DH5α_NDM-1_* transformant, which indicated that *bla*_TEM-1_ and *bla*_CTX-M-57_ were located on a different plasmid from *bla*_NDM-1_ (**Fig. 2**).

### MLST genotype analysis

MLST revealed that *E. coli BJ01* belonged to a new sequence type 224 (ST224).

### Genetic Environment of the *bla*_NDM-1_ gene

**Figure 4:**
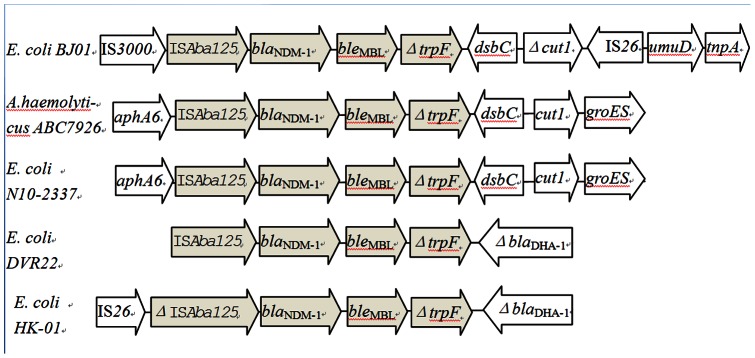
Analysis of the *bla*_NDM-1_ gene environment. Schematic drawing comparing the genetic elements surrounding the *bla*_NDM-1_ gene in *Escherichia coli BJ01*, *Acinetobacter haemolyticus ABC7926*(*GenBank* JQ080305), *E. coli N10-2337*(*GenBank* JF714412), *E. coli HK-01*(*GenBank* NC_019063), and *E. coli DVR22*(*GenBank* JF922606). Δ, truncated gene. The arrows indicate the orientation of each open reading frame. Similarity regions of the flanking region of NDM-1 gene between *BJ01* and other NDM-1-carrying isolates were labeled in grey color.

A 17,924-bp fragment was obtained from plasmid DNA of strain *E. coli BJ01*, and sequenced by primer walking. The sequence has been deposited in GenBank with accession No. JX296013. Sequencing of the *bla*_NDM-1_ upstream region identified the presence of an IS*Aba125* insertion sequence. Further sequence analysis revealed that the region flanking the IS*Aba125* was the IS*3000* gene. The *trpF* gene flanked the 3′ end of *bla*_NDM-1_, followed by *dsbC*, truncated *cut1* gene disrupted by IS*26*, *umuD* and a transposase gene. (**Fig. 4**)

## Discussion

In contrast to most other countries where NDM has been mainly identified in *Enterobacteriaceae*, in China to date NDM has only been reported in *Acinetobacter* [9], [11], [12], [24] and *Enterococcus faecium* [13]. However, other types of MBLs, such as IMP, and other classes of acquired carbapenemases, such a KPC, have been reported in Enterobacteriaceae in China[25]–[29], but isolates of NDM-1-producing *E.coli* have not previously been identified in China.

NDM-1-harboring strains could be highly multidrug resistant[3]. Previous reports on NDM-1-producing *Acinetobacter* spp. have demonstrated that strains resistant to all available antimicrobials except colistin were common in China[10], [30]. Our results showed resistant to almost all tested antimicrobials of the identified NDM-1-producing *E.coli* isolate. The patient had a good general condition and the infection was localized. Ultimately, the patient survived the infection. But this case should have raised public concerns again over increasing incidence of highly multidrug resistant NDM-1-harboring strains in China.

Travelers contribute significantly to the global movement of microbes and resistance genes [31]. Although nosocomial transmission of NDM-1 has occurred in many countries[26], [32], [33], medical tourism plays a significant role in the spread of NDM-1[34], and traveling to the Indian subcontinent is a significant risk factor of infection with an NDM-1-producing bacterium[35]. Patients in China who were found to have *bla*_NDM-1_-carrying bacteria had no history of traveling to the Indian subcontinent or another country[9], [11], [13], [24]. In this study, the patient carrying the *bla*_NDM-1_-positive *E. coli* strain had no foreign travel history.

The identified *BJ01* strain demonstrated phenotypes of both ESBL and NDM-1 (**Table 1**). This is consistent to previous reports on NDM-1-producers. For example, a study from China indicates that all 4 identified NDM-1-carrying *A. baumannii* isolates expressed both genes[30]. Data from the SMART study shows that *bla*_NDM-1_ positive enterobacteriaceae isolates often carry additional β-lactamase genes. Among 33 *bla*_NDM-1_-carrying strains, *bla*_CTX-M-15_ is detected in 30[36]. However, our data also suggested that ESBL and NDM-1 genes may be carried by distinct plasmids and could be transferred separately (**Fig. 1**). This has to be further confirmed by sequence analysis.

In our study, MLST analysis revealed that *BJ01* belonged to sequence type 224 (ST224), which was different from the NDM-1-producing ST101 *E. coli* isolated from Australia [37], Germany [38], Canada[39], the UK [40], and New Zealand [41]. This is the first reported ST224 *E.coli* strain producing NDM-1.

Analysis of the genetic environment of *bla*_NDM-1_ in *BJ01* reveals that the region flanking *bla*_NDM-1_ is very similar to some *Acinetobacter* spp. isolated in China [10], [11], [24] and *E. coli* plasmid pNDM102337 (GenBank: JF714412) isolated in Canada, in which *DsbC* and *cutI* are located downstream of the *bla*_NDM-1_. *cutI* is truncated by a transposon-like structure. In contrast with *E. coli* HK-01 (GenBank: HQ451074) and *E. coli* DVR22 (GenBank: JF922606.1), there was no *bla*_DHA_ downstream of *bla*_NDM-1_ in *BJ01*(**Fig. 4**). This finding indicates a potential link between *Acinetobacter* spp. and *Enterobacteriaceae* in term of the *bla*_NDM-1_ gene in China. More complex transposon-like structures are detected around *bla*_NDM-1_. IS*3000* and IS*Aba125* are located upstream of the *bla*_NDM-1_, while IS*26* and a transposase gene are located downstream of the *bla*_NDM-1_. Transposons should play an important role in horizontal transfer of the *bla*_NDM-1_ between different species of bacteria, such as from *Acinetobacter* spp. to *E. coli.*

In conclusion, in mainland China, this is the first reported case of infection due to a NDM-1-producing *E. coli*, which is highly drug-resistant and comes with a new MLST genotype in *E. coli*. The patient carrying this strain does not have a travel history The detection of the NDM-1-positive *E. coli* isolate in our study indicates immediate importance of strengthening surveillance to prevent the nosocomial infection and dissemination of NDM-1 in China.
